# Safety and Efficacy of Single-Stage Surgical Treatment for Congenital Scoliosis Associated with Intraspinal Mass

**DOI:** 10.1038/srep41229

**Published:** 2017-01-24

**Authors:** Bo-bo Zhang, Hui-ren Tao, Tai-lin Wu, Lin Wang, Chun-guang Duan, Tao Zhang, Tao Li, Wei-zhou Yang, Ming Liu, Jun Ma

**Affiliations:** 1 Department of Orthopaedics, Xijing Hospital, The Fourth Military Medical University, Xi’an 710032, China; 2 Department of Orthopaedics, The First Affiliated Hospital of Xi’an Jiaotong University, Xi’an 710061, China; 3 Department of Orthopaedics, The hospital of Xin Jiang production and construction corps, Urumqi 830002, China

## Abstract

For congenital scoliosis associated with intraspinal anomaly, surgical treatment is often advocated. However, the safety and efficacy of single-stage intraspinal mass resection and scoliosis correction remain unclear. The purpose of this study was to retrospectively evaluate the feasibility and risk factors of single-stage surgical treatment for congenital scoliosis associated with intraspinal mass. Patients’ clinical records were reviewed for demographic and radiographic data, operating time, intraoperative blood loss, perioperative complications, and postoperative pathologic results. Two female and 5 male patients with an average age of 19.14 ± 7.52 years (range, 11–31 years) were evaluated. Patients were followed for a minimum of 24 months after initial surgical treatment, with an average of 49.71 ± 32.90 months (range, 27–99 months). Spinal curvature was corrected from an average of 69.57 ± 20.44° to 29.14 ± 9.87°, demonstrating a mean correction rate of 55.05% ± 18.75%. No obvious loss of correction was observed at the final follow-up. Complications included transient neurologic deficit, cerebrospinal fluid leakage, and intraspinal mass recurrence in 1 patient each. There was no paralysis or permanent nerve damage. In conclusion, simultaneous intraspinal mass resection and scoliosis correction appears to be safe and effective.

Congenital scoliosis (CS) is the most common congenital deformity of the spine. This occurs most frequently in the first 8 weeks of prenatal development, during which time, the bony elements of the spine are forming, and the neuraxis is completing its infolding and closing of the neural tube^1^. These events are closely related, and any event that causes CS also may be associated with intraspinal anomaly, such as tethering of the cord, diastematomyelia, or intraspinal mass^2^. Severe scoliosis will disturb the growth of infants and adolescents, and may affect the heart, lungs, and spinal cord, causing paralysis. It is rare for CS to be associated with intraspinal mass^3^,^4^,^5^,^6^,^7^,^8^,^9^,^10^,^11^,^12^. The classic advocated approach in such patients is 2-stage surgery: first, for treatment of the intraspinal pathology, and, second, for correction and stabilisation of the deformity 3 to 6 months later^4^. Only one study has reported a patient who underwent simultaneous surgical treatment for congenital deformity and intraspinal mass and achieved a good outcome^11^. The present retrospective study was designed to evaluate the safety and efficacy of single-stage intraspinal mass resection and scoliosis correction with a minimum 2-year follow-up at a single centre.

## Materials and Methods

### Study Design

We evaluated 7 consecutive patients with CS associated with intraspinal mass who were treated with single-stage intraspinal mass resection and scoliosis correction from January 2007 to June 2016 at a single institution. The current study was approved by the Ethics Committee of The Fourth Military Medical University, also known as the Ethics Committee of Xijing Hospital. Written informed consent was obtained from all participants or their parents/guardians. The methods were carried out in accordance with approved guidelines. A minimum 2-year follow-up was required. Patients’ age, sex, clinical characteristics, radiologic data, operating details, postoperative pathologic results, perioperative complications, and surgical outcomes were evaluated. The effectiveness of the surgery was defined as significant correction of spinal curvature, while the safety of the surgery was defined as absence of postoperative paralysis or permanent nerve damage.

### Radiologic Examination

All patients underwent standing anteroposterior and lateral radiography of the spine from C1 to the sacrum, as well as supine right and left bending radiography. All patients also underwent 3-dimensional computed tomography and neural axis magnetic resonance imaging from the brainstem to the sacrum to detect associated intraspinal anomalies. Ultrasonography was performed as a screening tool to detect genitourinary anomalies. Cardiovascular evaluation including clinical examination and echocardiography was performed to exclude cardiac anomalies. Imaging measurements were evaluated by 2 independent observers. Cobb angles and coronal and sagittal alignment were measured on long cassette films using C7 as the plumb line reference. The change between pre- and postoperative Cobb angles was divided by the preoperative angle, and the result was designated as the correction rate.

### Data Collection and Follow-up

Clinical records and radiographic data of all patients were collected and analysed with regard to operating time, intraoperative blood loss, perioperative complications, and postoperative pathologic results. Postoperative radiographic data included amount of curvature correction immediately postoperatively and at the final follow-up. Patients were observed for a minimum of 24 months after initial surgical treatment. The muscle strength was graded as follows: Grade 0 = No contraction, Grade 1 = Trace of contraction, but no movement at the joint, Grade 2 = Movement at the joint with gravity eliminated, Grade 3 = Movement against gravity, but not against added resistance, Grade 4 = Movement against external resistance, but less than normal, Grade 5 = Normal strength.

### Surgical Technique

Halo-gravity traction was performed preoperatively in 1 patient with Cobb angles greater than 100°. Traction was continuous and transferable between bed and wheelchair. Initial traction force was 3 kg, while final traction force was equal to half of the patient’s body weight. The patient walked or exercised freely for 1 to 2 hours daily to reduce the potential risk of disuse osteoporosis. The patient underwent halo-gravity traction for 12 weeks with no complications.

All surgeries were performed under general anaesthesia. Patients were placed in the prone position with adequate padding for the chest and pelvis. Then, a midline incision was made. After exposure of determined levels and placement of instrumentation, careful laminectomy was performed from the cranial end to the caudal end of the intraspinal mass segment. Once the intraspinal mass was exposed, it was carefully resected. Then, instrumentation and correction of spinal curvature using a posterior fusion technique was performed. The overlying musculature, fascia, and skin were closed in anatomical layers. All patients were closely monitored intraoperatively according to both transcranial electric motor-evoked potential and somatosensory-evoked potential. Vertebral column resection and Smith-Petersen osteotomy were performed in 1 and 3 patients, respectively. After surgery, all patients were engaged in a supervised physical therapy program as well as a home exercise protocol.

### Data Analysis

Descriptive statistics, including means and SDs, were calculated for demographic and perioperative data. Comparisons between continuous variables were performed using the Student’s *t* test. Statistical analysis was performed using SPSS version 17.0 for Windows (SPSS Inc., Chicago, IL), with P values of less than 0.05 considered statistically significant.

## Results

Two female and 5 male patients with an average age of 19.14 ± 7.52 years (range, 11–31 years) were evaluated. Patients were observed for a minimum of 24 months after initial surgical treatment, with an average follow-up of 49.71 ± 32.90 months (range, 27–99 months). Average operating time was 550 ± 166 min (range, 370–855 min), and average blood loss was 2100 ± 663 mL (range, 1500–3000 mL). Postoperative pathologic examination revealed epidermoid cyst in 2 patients, dermoid cyst in 1, bronchogenic cyst in 1, teratoma in 2, and pilomyxoid astrocytoma in 1. All intraspinal masses were located at the thoracic segment. Five intraspinal masses were intramedullary, 1 was intradural extramedullary, and 1 was both intramedullary and intradural extramedullary (Table 1). There were no intraoperative neuromonitoring alerts. Four patients had neurologic deficits preoperatively. After surgery, 2 patients recovered to grade 5, 1 with paralysis in both lower limbs preoperatively recovered to grade 3 postoperatively, and 1 with right lower limb strength grade 1 preoperatively recovered to grade 3 postoperatively (Table 2).

The average coronal curve was 69.57 ± 20.44° (range, 45–101°) preoperatively, which was corrected to 29.14 ± 9.87° (range, 18–47°) immediately postoperatively, showing a correction rate of 55.05% ± 18.75%. At the final follow-up, the average coronal curve was 32.86 ± 10.45° (range, 21–54°). The average sagittal curve was 64.29 ± 33.01° (range, 33–115°) preoperatively, which was corrected to 34.57 ± 12.09° (range, 26–60°) immediately postoperatively, showing a correction rate of 41.53% ± 19.43%. At the final follow-up, the average sagittal curve was 37.29 ± 12.73° (range, 27–64°). There was no obvious loss of correction at the final follow-up (Table 3). Radiographic examination at the final follow-up showed good fixation and grafted bone fusion in all patients.

**Figure 1:**
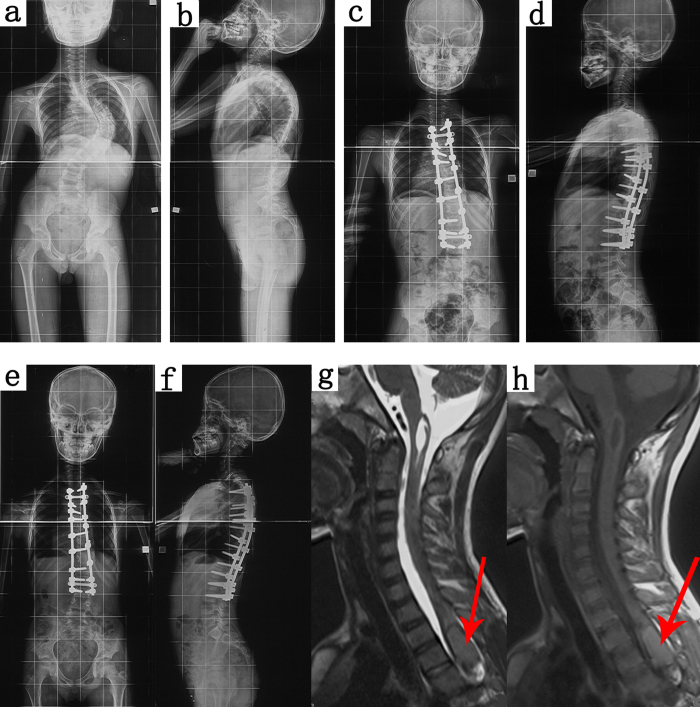
Images of an 11-year-old boy with congenital scoliosis associated with intraspinal mass. The patient underwent single-stage intraspinal mass resection and scoliosis correction. He experienced transient neurologic deficit postoperatively, but recovered to normal 6 months later. (**a,b**) Preoperative radiographs, demonstrating a left curve of 85° and kyphosis of 66°. (**c,d**) Postoperative radiographs, demonstrating a left curve of 18° and kyphosis of 36°. (**e,f**) Final follow-up radiographs, demonstrating a left curve of 26° and kyphosis of 42°. (**g,h**) Magnetic resonance images, demonstrating an intraspinal mass at T2–7.

All 7 patients underwent single-stage intraspinal mass resection and scoliosis correction. Postoperatively, 2 patients recovered to grade 5 and 2 showed improvement. However, at the 5-year follow-up, 1 patient with a dermoid cyst complained of numbness and weakness in the right lower limb for 1 month. Magnetic resonance imaging demonstrated intraspinal mass recurrence. After subsequent resection, the mass did not recur, and the patient demonstrated a good outcome. One patient with intramedullary pilocytic astrocytoma experienced transient neurologic deficit postoperatively (Fig. 1). Preoperatively, his spinal curvature was 85° and bilateral lower limb strength was grade 5. Total blood loss was 1900 mL and his intraoperative blood pressure was low. Thus, 6 units of suspended red blood cells and 840 mL of frozen plasma were transfused. His blood pressure stabilised and no adverse reactions occurred during transfusion. There was no change in his spinal cord evoked potential intraoperatively. After surgery, left lower limb strength decreased to grade 4. Five days after surgery, radiography showed good fixation and curvature correction. After 6 months, his muscle strength recovered to grade 5. One patient had cerebrospinal fluid leakage postoperatively, which was cured after 7 days of drainage. No patient had deep vein thrombosis, pulmonary embolism, infection, pneumothorax, pleural effusion, or pseudarthrosis.

## Discussion

Whether or not surgery is necessary depends on the natural progression of spinal curvature as well as on clinical and radiologic assessments. When choosing the appropriate surgical strategy, it is essential to detect the inherent spinal anomaly^2^. Intraspinal mass can cause progressive neural loss with growth and progression of curvature. In addition, their presence greatly increases the risk of neurologic injury during surgical correction of the deformity.

According to Winter *et al*.^13^. in patients with spinal deformity associated with intraspinal anomaly, the traditional approach is 2-stage surgery: first, for treatment of the intraspinal pathology, and, second, for correction and stabilisation of the deformity 3 to 6 months later. However, Hamzaoglu *et al*.^2^ reported that this 2-stage approach has certain drawbacks. First, because it comprises more than one surgery, the patient is exposed to the risks of surgery more than once. Difficult surgical exposure, increased bleeding, adhesion formation, and less clear anatomical landmarks make the subsequent corrective surgery more difficult. In addition, more complicated reconstructive procedures, such as osteotomy and hemivertebrectomy, may be difficult because of preformed adhesion at the surgical site. Since 2007, our management approach for these patients involves simultaneous surgery for the intraspinal pathology and congenital deformity via a posterior approach.

A total of 495 patients with CS have undergone surgical treatment in our department, 7 of whom had intraspinal mass, accounting for 1.41% of patients, which is similar to the 1.33% reported in previous literature^14^. In patients with CS associated with intraspinal mass, whether single- or 2-stage surgery is preferable remains controversial. In 2011, Abuzayed reported a patient with CS and intraspinal lipoma who underwent single-stage mass resection and scoliosis correction and achieved a good result^11^.

CS associated with intraspinal mass is extremely uncommon. We evaluated the safety and efficacy of simultaneous intraspinal mass resection and scoliosis correction via a posterior approach in such patients. In this study, the coronal curve correction rate was 55.05% ± 18.75%, which is similar to that reported in previous studies (48.1–66.0%) of patients who underwent scoliosis correction using a pedicle screw technique^15^,^16^. The coronal and sagittal curves were significantly corrected postoperatively (P = 0.001 and 0.032, respectively), and showed no obvious loss of correction at the final follow-up (P = 0.507 and 0.606, respectively). Based on these results, we can conclude that this single-stage surgery is effective.

Four patients had neurologic deficits preoperatively. After surgery, 2 recovered to grade 5 and 2 showed improvement. Two cases experienced complications: 1 with transient neurologic deficit and 1 with cerebrospinal fluid leakage. Regarding the patient with transient neurologic deficit, considering that his blood loss and intraoperative blood pressure were low, we considered that spinal cord ischemic injury might be the cause. There were no other complications during the follow-up period. Only 1 patient had recurrence, possibly due to continued secretion of residual epithelium or accumulation of cerebrospinal fluid in the residual cavity of the dermoid cyst, but, after subsequent surgical resection, he achieved a good outcome.

Our study is not without limitations. First, the present study was retrospective; thus, the evidence is not as compelling as that in prospective studies. Second, our study was conducted at a single centre with a limited number of cases. Finally, a direct comparison with 2-stage surgery was not performed because all consecutive cases underwent single-stage surgery at our centre. Further randomised multicentre studies are required to confirm the findings in the present study.

In conclusion, these patients who underwent single-stage surgery for CS associated with intraspinal mass had significant curvature correction without major complications. Based on our results, we can conclude that simultaneous intraspinal mass resection and scoliosis correction appears to be safe and effective.

## Additional Information

**How to cite this article**: Zhang, B.- *et al*. Safety and Efficacy of Single-Stage Surgical Treatment for Congenital Scoliosis Associated with Intraspinal Mass. *Sci. Rep.* **7**, 41229; doi: 10.1038/srep41229 (2017).

**Publisher's note:** Springer Nature remains neutral with regard to jurisdictional claims in published maps and institutional affiliations.

## Figures and Tables

**Table 1:** Patients’ clinical characteristics.

Case	Age, y	Sex	Follow-up time, mo	Operating time, min	Blood loss, mL	Location of intraspinal mass	Type of intraspinal mass
1	13	M	96	585	1600	Intramedullary (T5–7)	Teratoma
2	18	F	99	855	3000	Intramedullary (T1–3)	Epidermoid cyst
3	22	F	30	430	1500	Intradural extramedullary, intramedullary (T7–L1)	Teratoma
4	11	M	27	460	1900	Intramedullary (T3–9)	Pilocytic astrocytoma
5	13	M	30	490	1500	Intramedullary (T10)	Epidermoid cyst
6	31	M	39	665	2200	Intradural extramedullary (T5–10)	Bronchogenic cyst
7	26	M	27	370	3000	Intramedullary (T1–3)	Dermoid cyst

**Table 2:** Patients’ neurologic status.

Case	Preoperative	Postoperative	Complications
1	Weakness in right lower limb (grade 1)	Recovery to grade 3	None
2	Pain and numbness in right lower limb	Recovery to normal	Recurrence at 5-year follow-up
3	Paralysis in both lower limbs (grade 0)	Recovery to grade 3	Cerebrospinal fluid leakage
4	Normal	Strength of left lower limb decreased to grade 4	Transient strength decrease, recovery to grade 5 six months later
5	Normal	Normal	None
6	Weakness and numbness in both lower limbs (grade 4)	Recovery to grade 5	None
7	Normal	Normal	None

**Table 3:** Changes in coronal and sagittal Cobb angles.

	Preoperative	Postoperative	Final follow-up	P1	P2
Coronal Cobb angle°	69.57 ± 20.44	29.14 ± 9.87	32.86 ± 10.45	0.001	0.507
Sagittal Cobb angle°	65.14 ± 31.91	33.71 ± 12.51	37.29 ± 12.72	0.032	0.606

Data are presented as mean ± SD. P1 refers to change between pre- and postoperative. P2 refers to change between postoperative and final follow-up.
